# Novel mutations in *PANK2* and *PLA2G6* genes in patients with neurodegenerative disorders: two case reports

**DOI:** 10.1186/s12881-017-0439-y

**Published:** 2017-08-18

**Authors:** Hassan Dastsooz, Hamid Nemati, Mohammad Ali Farazi Fard, Majid Fardaei, Mohammad Ali Faghihi

**Affiliations:** 1 Comprehensive Medical Genetic Center, Shiraz University of Medical Sciences, Shiraz, Iran; 2 Shiraz Neuroscience Research Center, Shiraz University of Medical Sciences, Shiraz, Iran; 3 Department of Medical Genetics, Medical School, Shiraz University of Medical Sciences, Shiraz, Iran; 4 Center for Therapeutic Innovation and Department of Psychiatry and Behavioral Sciences, University of Miami Miller School of Medicine, Miami, USA

**Keywords:** PLA2G6, PKAN, NBIA, *PANK2*, Case report

## Abstract

**Background:**

Neurodegeneration with brain iron accumulation (NBIA) is a genetically heterogeneous group of disorders associated with progressive impairment of movement, vision, and cognition. The disease is initially diagnosed on the basis of changes in brain magnetic resonance imaging which indicate an abnormal brain iron accumulation in the basal ganglia. However, the diagnosis of specific types should be based on both clinical findings and molecular genetic testing for genes associated with different types of NBIA, including *PANK2, PLA2G6, C19orf12, FA2H, ATP13A2, WDR45, COASY, FTL, CP,* and *DCAF17.* The purpose of this study was to investigate disease-causing mutations in two patients with distinct NBIA disorders.

**Case presentation:**

Whole Exome sequencing using Next Generation Illumina Sequencing was used to enrich all exons of protein-coding genes as well as some other important genomic regions in these two affected patients. A deleterious homozygous four-nucleotide deletion causing frameshift deletion in *PANK2* gene (c.1426_1429delATGA, p.M476 fs) was identified in an 8 years old girl with dystonia, bone fracture, muscle rigidity, abnormal movement, lack of coordination and chorea. In addition, our study revealed a novel missense mutation in *PLA2G6* gene (c.3G > T:p.M1I) in one and half-year-old boy with muscle weakness and neurodevelopmental regression (speech, motor and cognition). The novel mutations were also confirmed by Sanger sequencing in the proband and their parents.

**Conclusions:**

Current study uncovered two rare novel mutations in *PANK2* and *PLA2G6* genes in patients with NBIA disorder and such studies may help to conduct genetic counseling and prenatal diagnosis more accurately for individuals at the high risk of these types of disorders.

## Background

Neurodegeneration with brain iron accumulation (NBIA) is etiologically and clinically a heterogeneous group of inherited neurological disorders characterized by basal ganglia iron deposition, mainly in the globus pallidus and/or substantia nigra. The hallmark of NBIA include dystonia, dysarthria, spasticity, and Parkinsonism [1–4]. However, apart from these neurological manifestations and neuropathological findings, other abnormalities like retinal degeneration and optic atrophy are common in patients with NBIA [3, 4]. Up to now, the genetic basis of 10 types of NBIA has been established which include Aceruloplasminemia [5], Beta-propeller protein-associated neurodegeneration [6], COASY protein-associated neurodegeneration [7, 8], Fatty acid hydroxylase-associated neurodegeneration [9], Kufor-Rakeb syndrome [10], mitochondrial membrane protein-associated neurodegeneration [11], Neuroferritinopathy [12, 13], *PLA2G6*-associated neurodegeneration (PLAN) [14, 15], Pantothenate kinase-associated neurodegeneration (PKAN) [16], and Woodhouse-Sakati syndrome [17]. It has been reported that the major percentage of NBIA is attributed to autosomal recessive mutations in Pantothenate Kinase 2 (*PANK2*) gene [18], which is resulted in PKAN [16], and Phospholipase A2 Group VI (*PLA2G6*) gene, leading to PLAN [19].

PKAN is divided into two types which include classic PKAN, with early onset in the first decade of life and rapid progression, and atypical PKAN with rare, later onset and slower progression [18]. Children with PKAN have typically gait difficulties approximately at the age of three and at later life they usually show progressive dystonia, rigidity, dysarthria, and spasticity. However, patients with later-onset PKAN present speech difficulty and psychiatric symptoms [20, 21]. It is worth noting that in individuals with PKAN, Magnetic Resonance Imaging (MRI) is characterized by “eye-of-the-tiger” sign, T2-hypointensity of the globus pallidus with a central hyperintensity, corresponding to excessive brain iron accumulation [22] and predicting a disease causing mutation in *PANK2* gene [23]. However, mutation detection is a gold standard to confirm diagnosis in a patient even if the radiologic findings show the typical eye-of-the-tiger sign since there is no a strong correlation between this sign and *PANK2* mutations. Another main form of NBIA is PLAN which is caused by mutation in *PLA2G6* gene. PLAN is characterized by three phenotypes, including infantile neuroaxonal dystrophy (INAD), atypical neuroaxonal dystrophy (NAD), and *PLA2G6-*related dystonia-parkinsonism [24, 25]. INAD phenotype which is occurred between ages 6 months and 3 years is usually manifested with developmental regression, progressive psychomotor delay, initial hypotonia and progressive spastic tetraparesis. Regarding the atypical NAD which is presented later in childhood, it is commonly observed with slower progression, dystonia, spastic tetraparesis, speech delay and diminished social interactions [26–28]. By contrast, *PLA2G6-*related dystonia-parkinsonism is manifested in late adolescence/early adulthood with marked cognitive decline, pyramidal tract signs, and eye movement abnormalities. It should be noted that in the brain MRI, the hallmark features of both INAD and atypical NAD are recognized as cerebellar atrophy and optic atrophy, and in more cases brain iron accumulation is usually detected in the globus pallidus [25, 29].

By the fact that up to now various genes (*PANK2*, *PLA2G6*, *C19orf12*, *FA2H*, *ATP13A2*, *WDR45*, *COASY*, *FTL*, *CP*, and *DCAF17* [30]*)* have been shown to be associated with different types of NBIA and other neurodegenerative disorders, the aim of this study was to investigate disease-causing mutations using Next Generation Sequencing (NGS) method in two patients with neuromuscular and neurodegenerative disorders.

## Case presentation

Here we report two Iranian and Afghan patients born in consanguineous families affected by NBIA. The diagnosis was made on the basis of the clinical findings of a progressive movement disorder.

### Family I, patient I

An 8- year- old Iranian girl was admitted to Namazi Hospital (Shiraz, Iran) in 2015 with clinical diagnosis of dystonia. She was apparently normal before the age of 4 years but after that she developed bone fracture, muscle rigidity, abnormal movement, lack of coordination, chorea, and dystonia with seizure attacks. She was intellectually normal but she had speech problem due to the use of medications including Sirdalud (Tizanidine), Gabax, trihexidine, and NA Valporate.

**Fig. 1:**
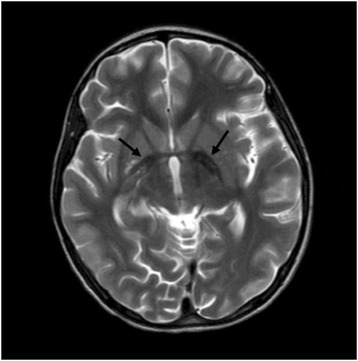
MRI features in patient with PKAN. T2-weighted brain MRI of the 8-year-old patient shows bilateral symmetrical hypointensity in the globus pallidus with central hyperintensity, giving an eye-of-the-tiger sign (arrows)

Multiplanar multisequential MRI were taken through the brain with usual protocol which demonstrated normal signal intensity of both cerebral hemispheres with no sign of mass, hemorrhage, and ischemic infarction. Hydrocephalus and shift of midline structure were not found. Posterior fossa structures including cerebral hemispheres showed normal signal intensity without any mass, hemorrhage, and ischemic infarction. 7the-8the nerve root complexes appeared normal and pituitary gland was also normal without the sign of gross mass. Also, extra-axial mass, hematoma, and fluid collection were not observed. It is worth noting that generalized cortical atrophy was considerable which was more than that of expected for the patient’s age. In addition, mucosal thickening was noted at both ethmoidal maxillary sinuses due to sinusitis. Moreover, mild inflammatory change at right mastoid air cells and the “eye-of-the-tiger” sign in MRI were remarkable (Fig. 1). But, M.R.I of the cervical spine without contrast showed normal features. Paraclinical examinations were also requested which showed increased level of alkaline phosphatase (ALP) (191 U/L) and creatine phosphokinase (CPK) (456 U/L).

The proband died at the age of 9 years with the severe abnormalities mentioned above. Now, her family would arbitrarily prefer to use the identified mutation for prenatal diagnosis which may help them to have a healthy child.

### Family II, patient II

One and half- year-old boy from Afghanistan with muscle weakness at the onset of disease (a case of neuromuscular disease) was admitted to comprehensive children's development in Emam Reza Hospital (Shiraz, Iran) in 2014. Diagnostic evaluations were brain MRI and abdominal and pelvic ultrasonography. There were no intellectual impairments and hepatosplenomegaly at the age of one and half year. At the age of two, he showed neurodevelopmental regression (speech, motor and cognition) and floppy infant (hypotonia) but there were no deep tendon reflexes (DTR) and seizure. The ultrasonography showed normal features but MRI revealed only a minimal change of periventricular white mater which could be due to mild delayed myelination. Up to now, he has not been on any treatments. Two of his sisters died at the age of 4 and 6 years with similar phenotypes but with more severe neurodevelopmental abnormalities starting at the age of 8 months, in which they were not able to speak completely and they could not cry with any voices but only it could be recognized with tears on their eyes.

Comprehensive laboratory examinations were also requested, including hematology, biochemistry, hormone, and urine analysis. The positive and abnormal findings for this patient were the decreased level of hemoglobin (Hb) (11.8 g/dL), hematocrit (HCT) (34.5%), mean corpuscular volume (MCV) (68.73 fL), mean corpuscular hemoglobin (MCH) (23.51 pg), and increased level of CPK (1124 U/L), lactate dehydrogenase (LDH) (542 μ/L), and aspartate aminotransferase (AST, SGOT) (64 U/L) enzymes. In addition, genetic tests for Spinal Muscular Atrophy (SMA) and Duchenne Muscular Dysrtrophy (DMD) disease showed negative results and therefore Whole Exom Sequencing (WES) was suggested to the family.

WES was utilized for amplification and sequencing of all exons of protein-coding genes as well as some of other important genomic regions. The DNA samples were sequenced, using Illumina HiSeq2000 machine and standard Illumina protocol for pair-end 99-nucleotide sequencing. WES detail of coverage and number of reads are listed in Table 1. Briefly, NGS was performed to sequence close to 100 million reads on Illumina HiSeq2000 Sequencer. In general, test platform examined >95% of the targeted regions with sensitivity of above 99%. In this test, point mutations and micro-insertion/deletions and duplication (<20 bp) can be simultaneously detected. Bioinformatics analysis of the sequencing results was performed using BWA aligner [31], GATK [32] and annovar [33] open access software as well as public databases and standard bioinformatics software such as CADD-Phred, SIFT, PolyPhen, GERP, PhastCons, LRT, Mutation Assessor, Mutation Taster, and other programs.

**Table 1:** Whole Exome Sequencing detail of coverage and number of reads

Type	Value	Type	Value
Number of mapped reads	41,674,840	Percent reads on target	95.70%
Number of amplicons	293,903	Total assigned amplicon reads	39,882,524
Percent assigned amplicon reads	95.70%	Average reads per amplicon	136
Uniformity of amplicon coverage	86.30%	Amplicons with at least 100 reads	53.69%
Amplicons with at least 1 read	99.54%	Amplicons with at least 500 reads	0.70%
Amplicons with at least 20 reads	90.02%	Amplicons reading end-to-end	35.97%
Amplicons with no strand bias	85.64%	Total aligned base reads	7,342,243,527
Bases in target regions	57,742,646	Total base reads on target	6,979,820,754
Percent base reads on target	0.95	Uniformity of base coverage	0.85
Average base coverage depth	121	Target bases with no strand bias	78.31%
Target base coverage at 1×	99.18%	Target base coverage at 100×	47.95%
Target base coverage at 20×	87.91%	Target base coverage at 500×	0.62%
Percent end-to-end reads	58.98%	mapping rate	99.10%
AQ17	92.21%	AQ20	87.51%

For confirmation of novel mutations, whole blood samples from family members of the probands were collected in EDTA tubes and then genomic DNA was extracted from the peripheral blood lymphocytes by QIAamp DNA Blood Mini Kit (Germany) according to the manufacturer’s instructions. After that, the genomic DNA concentration was measured by NanoDrop (ND1000, USA) and stored at −20 °C until use. PCR was then performed for the probands and their parents using following primers: F-PANK2:GTGTTGTCCTGGAACTGTCTG and R-PANK2 CCCACCCCAAATGACTACATTTA (PCR product: 563 bp) to amplify exon 5 of PANK2 and F-PLA2G6*:* GCCAATAAGACCTCCAATC and R-PLA2G6*:* GTCACTTTTACCTCCCACTC (PCR product: 515 bp) to amplify exon 2 of *PLA2G6.* Then, amplified DNA was subjected to Sanger sequencing using both forward and reverse primers according to ABI BigDye Terminator Cycle Sequencing Kit (Applied Biosystems®, USA). Sanger sequencing data was analyzed using NCBI BLAST and CodonCode Aligner software. Multiple sequence alignment analysis extracted from Polyphen website was also used to compare the amino acid sequence of human PANK2 and PLA2G6 proteins with corresponding proteins across all Kingdoms. Following bioinformatics software and websites were also used to identify the features of PANK2 and PLA2G6 and the consequences of mutations in the given position of the proteins: Polyphen, Mutation Taster, SIFT, STRING software (search tool for the Retrieval of Interacting Genes/Proteins: string.embl.de/) and DISOPRED3 (Intrinsic disorder predictor).

**Fig. 2:**
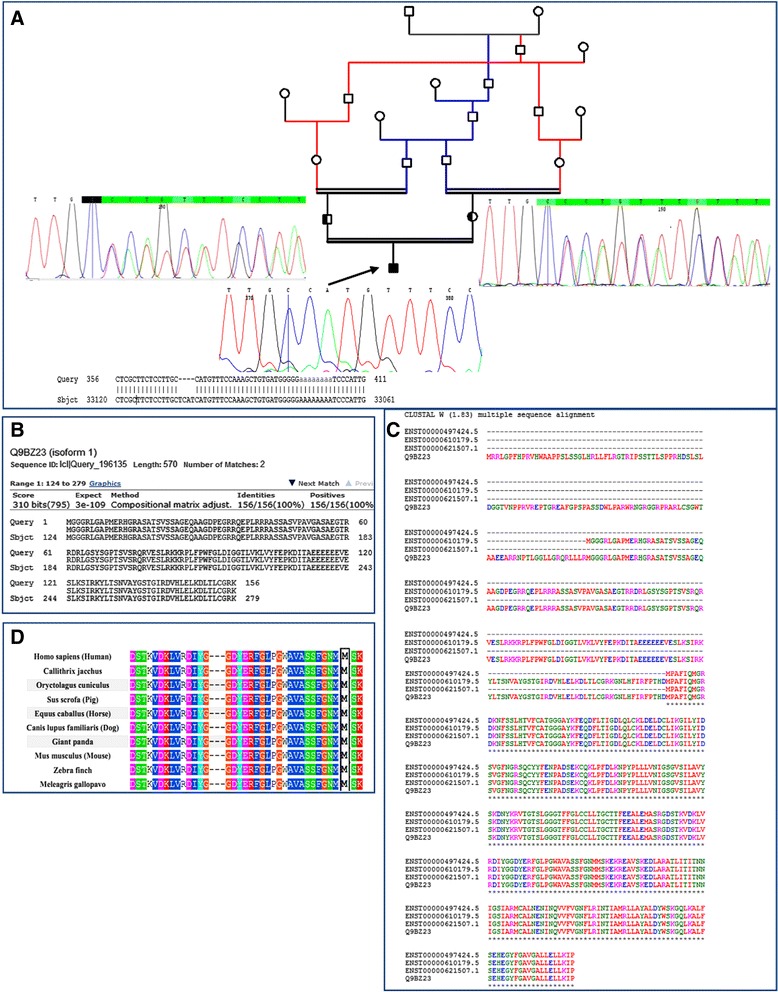
Confirmation of new mutation in family I. **a** Using Sanger sequencing, the inheritance mode of autosomal recessive was confirmed in this family on the basis of identified heterozygote mutation in parents and homozygote in the proband. **b** PANK2 transcript leading to Nonsense mediated decay. **c** Multiple sequence alignment of all human encoding isoforms of PANK2 using Clustal W which shows the same conserved residues in these isoforms. **d** Comparative amino acids alignment of PANK2 protein across all Kingdoms

Sequences text files obtained from WES were aligned using BWA aligner tool and variants were identified using GATK and annotated utilizing annovar software. In total, more than 120 K annotated variants were identified with hetero/homo ratio of 1.6 to 1.8, which then were filtered based on their frequency, location, functional consequences, inheritance pattern, and more importantly clinical phenotype. In family I, a novel deleterious homozygous four-nucleotide deletion causing frameshift mutation (NM_153638: exon 5, c.1426_1429delATGA, p.M476 fs) was identified in *PANK2* gene. Mutations and small deletions in *PANK2* gene have been reported in patients with NBIA1(OMIM: 234,200). The disease is also called PKAN and apparently causes dystonia in affected individuals. Regarding the family II, a novel deleterious homozygous missense mutation was found in *PLA2G6* gene (NM_001004426: exon 2: c.3 G > T: p.M1I). These identified mutations were not reported before and therefore, are classified as the variants of unknown significance (VUS). Following evidences can confirm that this *PANK2* mutation results in PKAN: **1-** WES using NGS revealed only this mutation to be the cause of PANK in the patient. **2-** As shown in Fig. 2a, using Sanger sequencing, the mutation was confirmed in the proband and the inheritance pattern based on heterozygote mutation identified in her parents must be an autosomal recessive mode. **3-**This four-nucleotide deletion (c.1426_1429delATGA) causes frameshift after codon 476 in PANK2 protein, leading to the premature translation termination which can make it highly likely to contribute to the observed phenotype in the patient. 4- Despite the mutation is in the 3′ end of the open reading frame of this protein, it is predicted that it can produce a completely nonfunctional truncated polypeptide since one of the reported transcript for this gene (ENST00000336066.7, V9GYZ0) with the absence of all amino acids after position 279 is resulted in the nonsense mediated decay (Fig. 2b). Inaddition, using Clustal W Multiple Sequence Alignment (Fig. 2c), it can be seen that after codon 191 all amino acid are included in all functional isoforms of PANK2, representing the vital presence of these codons in the protein. **5-** This mutation is close to similar mutations in *PANK2* gene that have been reported to cause NBIA in the basal ganglia of the brain. **6-** According to Mutation Taster online software, this variation is predicted to be a disease causing variant **7-** The comparative amino acids alignment of PANK2 protein across all Kingdoms was also performed using multiple sequence alignment analysis extracted from Polyphen website and, as shown in Fig. 2d, residue in this region is highly conserved during evolution. As a result, these evidences can prove that this deletion mutation in *PANK2* gene can be the genetic cause of PANK in family I.

**Fig. 3:**
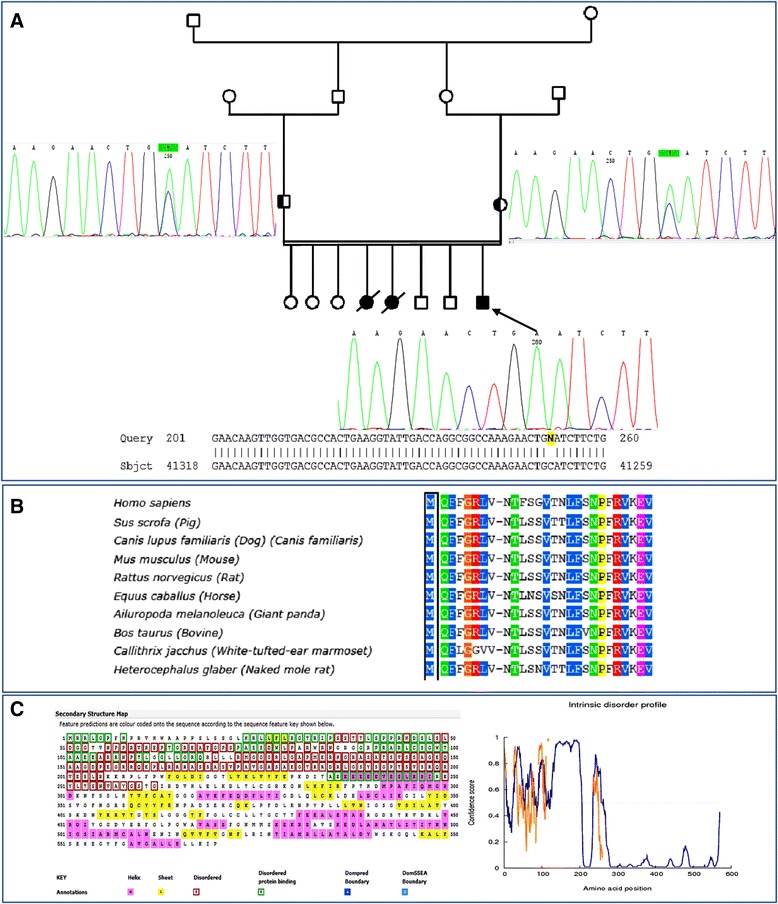
Confirmation of novel mutation in family II. **a** Confirmation of autosomal recessive pattern of *PLA2G6* mutation in the proband with PLAN disorder. **b** Comparative amino acids alignment of PLA2G6 protein across all Kingdoms. **c** Intrinsic disorder profile for PLA2G6 and its secondary structure map predicted by DISOPRED3. Amino acids in the input sequence are considered disordered when the dark line is above the gray dashed line, that is the confidence score is higher than 0.5

Regarding the *PLA2G6,* following evidences can prove that its mutation in our patient results in PLAN: **1-** c.3 G > T mutation is caused the first codon, ATG, to be shifted, leading to abnormal protein and making it highly likely to contribute to the observed phenotype in the patient. **2-** This mutation is close to similar mutation in first codon of *PLA2G6* gene (Met1Val) [28] that has been reported to lead to the NBIA (INAD1 form) **3-** WES identified only this mutation to be the main cause of PLAN in the patient. **4-** As shown in Fig. 3a, using Sanger sequencing, the mutation was confirmed in the proband and on the basis of identified heterozygote mutation in his parents, the inheritance pattern must be an autosomal recessive mode. **5-** Mutation Taster, SIFT, and Polyphen online software predicted that this variation will be damaging **6-** As can be seen in Fig. 3b, the comparative amino acids alignment of PLA2G6 protein across all Kingdoms using multiple sequence alignment analysis extracted from Polyphen website showed that this residue is highly conserved during evolution. **7**- Intrinsic disorder profile for PLA2G6 predicted by DISOPRED3 revealed that amino acids in some region of protein including the first amino acids are considered disordered when the dark line is above the gray dashed line (Fig. 3c). This amino acids are also involved in protein binding and, therefore they are very important in its functional state (Fig. 3c). As a result, this mutation in *PLA2G6* gene can lead to the PLAN in the family II.

## Discussion

**Fig. 4:**
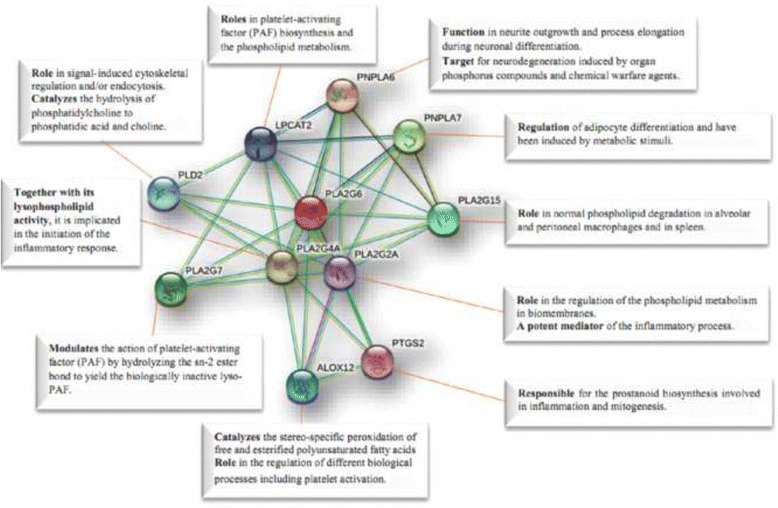
Possible interactions between PLA2G6 and other proteins using STRING software. It shows that these interactions may involve in different features of NBIA diseases

**Fig. 5:**
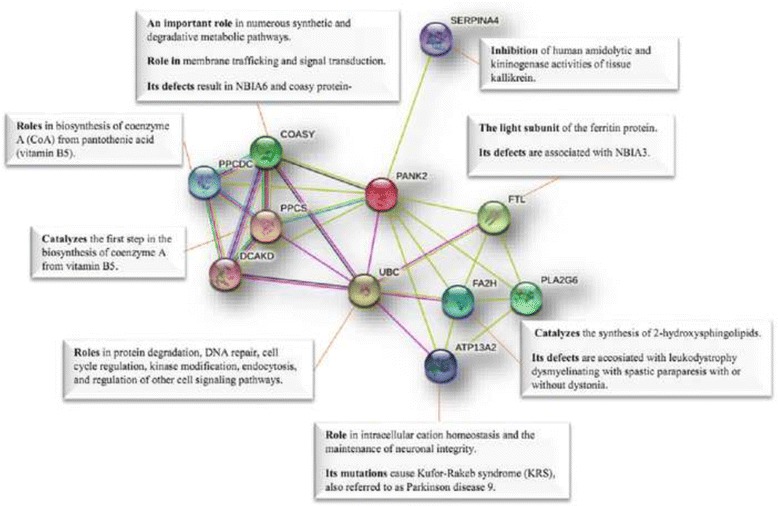
Possible interactions between PANK2 and other proteins using STRING software. It reveals that these possible associations may involve in different characteristics of NBIA disorders

To identify that possible interactions between PLA2G6 and PANK2 proteins and other partners may play important roles in pathogenesis of NBIA and other neurodegenerative disorders, we used STRING software and as shown in Figs. 4 and 5, several predicted functional partners interacting PLA2G6 and PANK2 were identified. It worth noting that these two protein are also predicted to have an interaction with each other and therefore they may have roles in the same complex protein network involved in Iron metabolism.

Pantothenate kinase which is a ubiquitous and major cofactor in all organisms plays a central role (as an essential regulatory enzyme) in the metabolism of carboxylic acids, such as coenzyme A (CoA). It catalyzes the first and rate limiting step in the universal five-step CoA biosynthesis pathway and its activity is primarily regulated through feedback inhibition by acyl CoA species [34–36]. Up to now, three distinct types of pantothenate kinase enzymes have been identified which include type I (a prokaryotic PanK that predominates in eubacteria), type II (mainly in eukaryotic organisms), and type III (with a wider phylogenic distribution) [37].

PANK2 which appears to be the only mitochondria-targeted human PanK is involved in a myriad of metabolic reactions, including metabolism of water-soluble vitamins (such as B5) and cofactors [38]. This gene located on chromosome 20 (20p13) consists of 7 exons [16] and its different isoforms are generated by alternative PANK2 mRNA splicing with the use of alternate first exons. But, as reported in literature, only two PanK2 protein isoforms are proteolytically produced to form a mitochondrially localized, mature PanK2 [39]. Mutations in these isoforms are associated with HARP syndrome and PKAN, formerly Hallervorden-Spatz syndrome. Approximately 100 mutations in *PANK2* have been found in affected individuals with PKAN [16, 40–42]. The most common *PANK2* mutations are G411R and T418 M accounted for one-third of the disease alleles [16]. Usually patients with the severe early-onset form of the disorder have *PANK2* mutations that resulted in the complete absence of functional PANK2 [43]. But, the disease in cases affected by the later-onset form is typically resulted from changes of single amino acids in the enzyme, producing a protein retaining some functional properties [18, 44]. So, the residual activity of PANK2 in mitochondria determines the age of disease onset and it is proposed to be the best indicator of clinical findings [44]. It is well recognized that PKAN symptoms (classic PKAN) are usually manifested in early childhood while atypical PKAN is referred to the condition presented in teenage life. According to our data, onset in our PANK2-positive patient was 4 years and, therefore this case can be classified as “classic PKAN”. This patient was homozygous for *PANK2* deletion mutation at position c.1426_1429delATGA, p.M476 fs. This mutation has not been previously reported and may be associated with early onset and rapid progression disease. PLA2G6, Calcium-Independent Phospholipase A2 Group VI, which catalyzes the release of fatty acids from phospholipids may have a role in normal phospholipid remodeling, vasopressin-induced arachidonic acid release, leukotriene and prostaglandin production, fas-mediated apoptosis, and transmembrane ion flux in glucose-stimulated B-cells [45]. *PLA2G6* located on 22q13.1 consists of 17 exons which is subjected to transcription of several encoding isoforms but until now, only the features of its three full-length transcripts have been reported. Abnormal function of this PLA2 group VI enzyme may impair the integrity of cell membrane, leading to several neurodegenerative disorders [24, 25]. It has been found that various mutations in *PLA2G6* are associated with Parkinson disease 14 (PARK14, MIM:612,953) [46], autosomal recessive form of INAD1(MIM:256600) [24, 28], Neurodegeneration with brain iron accumulation 2A (NBIA2A, MIM: 256,600) and 2B (NBIA2B, MIM: 610,217) [24, 27].

PARK14 which is a progressive neurodegenerative disorder with an adult onset is characterized by parkinsonism, dystonia, severe cognitive decline, cerebral and cerebellar atrophy, and absence of iron in the basal ganglia on MRI [46]. Regarding the NBIA2A, it is a neurodegenerative disease characterized by the unique pathological feature of NAD, including axonal swelling and spheroid bodies in the central nervous system. The typical symptoms of the disease is started in the first 2 years of life and is finally led to the death around the age of 10 years. In relation to the NBIA2B, it is a neurodegenerative disorder with iron accumulation in the brain, primarily in the basal ganglia, and is characterized by progressive extrapyramidal dysfunction leading to rigidity, dysarthria, sensorimotor impairment, and dystonia [24, 27]. Concerning the INAD, it is a rare autosomal recessive neurodegenerative disorder with axonal swell and high levels of brain iron resulting to the intellectual disability and movement problems. At least 50 mutations in the *PLA2G6* gene have been identified in cases with INAD [24, 28]. In our study a novel homozygous mutation in *PLA2G6* gene (c.G3 T:p.M1I) was identified in an Afghan patient with INAD phenotype (due to the age of the disease onset, 1.5 year, and manifestations of developmental regression and progressive psychomotor delay).

To understand the pathomechanism of PLAN and PKAN characterized by degenerative changes of neuronal tissues, it is essential to identify the *PANK2* and *PLA2G6* mutations. It has been shown that different mutations in *PLA2G6* and *PANK2* are caused distinct neurological disorders with a heterogeneity of phenotypes and a variable age of disease onset, which may be due to disrupted interactions between these proteins and their predicted partners in a complex protein network. Up to now, no drugs have been used to treat the disorder, and the initial step in drug discovery research is finding out essential proteins or drug targets for a biological process. Using STRING software diferent possible protein partners were found in our study and understanding the exact mechanism of these predicted proteins and pathways may shed light into the therapeutic strategies for NBIA and related neurodegenerative disorders with the use of these proteins (through their up or down regulation) or any known drugs.

## Conclusions

Two rare novel mutations in *PANK2* and *PLA2G6* genes were identified in our patients with neuromuscular and NBIA disorders and such studies may help to conduct genetic counseling and prenatal diagnosis more accurately for individuals at the high risk of these disorders.

## Abbreviations

ALP: Alkaline phosphatase
AST: Aspartate aminotransferase
CoA: Coenzyme A
CPK: Creatine phosphokinase
DTR: Deep tendon reflexes
Hb: Hemoglobin
HCT: Hematocrit
INAD: Infantile neuroaxonal dystrophy
LDH: Lactate dehydrogenase
MCH: Mean corpuscular hemoglobin
MCV: Mean corpuscular volume
MRI: Magnetic resonance imaging
NAD: Neuroaxonal dystrophy
NBIA: Neurodegeneration with brain iron accumulation
NGS: Next generation sequencing
PKAN: Pantothenate kinase-associated neurodegeneration
PLAN: PLA2G6-associated neurodegeneration
S.O.L: Space-occupying lesion
WES: Whole exome sequencing
